# Development of Subject-Specific Proximal Femur Finite Element Models Of Older Adults with Obesity to Evaluate the Effects of Weight Loss on Bone Strength

**DOI:** 10.4172/2329-9509.1000213

**Published:** 2018-03-08

**Authors:** SL Schoell, AA Weaver, DP Beavers, Leon Lenchik, AP Marsh, WJ Rejeski, JD Stitzel, KM Beavers

**Affiliations:** 1 Department of Biomedical Engineering, Wake Forest School of Medicine, USA; 2 Department of Biostatistical Sciences, Wake Forest School of Medicine, USA; 3 Department of Radiology, Wake Forest School of Medicine, USA; 4 Department of Health and Exercise Science, Wake Forest University, USA

**Keywords:** Biomechanics, Proximal femur strength, Bone QCT, Bioengineering, FEA, Weight loss

## Abstract

**Study background:**

Recommendation of intentional weight loss in older adults remains controversial, due in part to the loss of bone mineral density (BMD) known to accompany weight loss. While finite element (FE) models have been used to assess bone strength, these methods have not been used to study the effects of weight loss. The purpose of this study is to develop subject-specific FE models of the proximal femur and study the effect of intentional weight loss on bone strength.

**Methods:**

Computed tomography (CT) scans of the proximal femur of 25 overweight and obese (mean BMI=29.7 ± 4.0 kg/m^2^), older adults (mean age=65.6 ± 4.1 years) undergoing an 18-month intentional weight loss intervention were obtained at baseline and post-intervention. Measures of volumetric BMD (vBMD) and variable cortical thickness were derived from each subject CT scan and directly mapped to baseline and post-intervention models. Subject-specific FE models were developed using morphing techniques. Bone strength was estimated through simulation of a single-limb stance and sideways fall configuration.

**Results:**

After weight loss intervention, there were significant decreases from baseline to 18 months in vBMD (total hip: −0.024 ± 0.013 g/cm^3^; femoral neck: −0.012 ± 0.014 g/cm^3^), cortical thickness (total hip: −0.044 ± 0.032 mm; femoral neck: −0.026 ± 0.039 mm), and estimated strength (stance: −0.15 ± 0.12 kN; fall: −0.04 ± 0.06 kN). Adjusting for baseline bone measures, body mass, and gender, correlations were found between weight change and change in total hip and femoral neck cortical thickness (all p<0.05). For every 1 kilogram of body mass lost cortical thickness in the total hip and femoral neck decreased by 0.003 mm and 0.004 mm, respectively. No significant correlations were present for the vBMD or strength data.

**Conclusion:**

The developed subject-specific FE models could be used to better understand the effects of intentional weight loss on bone health.

## Introduction

In addition to age, weight loss is a well-recognized risk factor for fracture. In seminal epidemiologic papers by Ensrud et al., authors repeatedly demonstrate that weight loss in older adults is associated with a near doubling of fracture risk, even after accounting for baseline body mass index (BMI) and weight loss intentionality [1,2]. The increase in fracture risk is partly attributed to a loss in areal bone mineral density (aBMD) as measured by dual x-ray absorptiometry (DXA). The effect of weight loss in older adults on other bone measures, such as cortical thickness and bone strength, has not been adequately investigated [3]. Given the limitations of DXA in predicting all fracture risk [4] and measuring BMD in the context of obesity and weight loss [5,6], assessment of additional bone measures in the context of older adults undergoing intentional weight loss would contribute to the knowledge and understanding of the mechanism underlying the deleterious effect of weight loss on bone health.

Unlike DXA, quantitative computed tomography (QCT) allows for measurement of volumetric bone mineral density (vBMD) and cortical thickness. Cortical thickness and its distribution across the proximal femur is an important factor in bone strength and fracture risk as patients with femoral neck fractures tend to have thinner cortices compared to age and sex-matched controls [7,8]. vBMD has been shown to predict fracture risk as well as hip aBMD in postmenopausal women and older men [9,10]. However, both aBMD and vBMD do not fully explain the ability of bone to resist physical forces. In evaluating bone strength, aBMD and vBMD only account for 56-72% and 47-87% of the variance in proximal femoral bone strength, respectively [11,12]. To improve the assessment of bone strength and hip fracture risk, subject-specific finite element (FE) models have been used to non-destructively estimate bone strength *in vivo* [11,13–18]. CT-based subject-specific FE models based on cadaveric subjects have shown better predictions of bone strength, compared to BMD measures derived from either DXA or QCT [11]. The advantage of subject-specific FE models is the ability to account for variations in geometry, cortical thickness, and material properties which all contribute to bone strength. Existing subject-specific proximal femur FE models are typically voxel-based and do not account for the distinction between cortical and trabecular bone to accurately model cortical thickness and compartment-specific material properties. While FE analysis of CT data is a well-established technique to assess bone strength, no study has assessed the changes in femoral bone strength in older adults undergoing intentional weight loss.

Therefore, the primary purpose of this study is to develop subject-specific proximal femur FE models using morphing techniques to examine the effect of intentional weight loss over 18 months in obese, older adults on bone measures including vBMD, cortical thickness, and bone strength derived from CT scan data of the total hip and femoral neck. Secondarily, we assessed baseline correlations between participant demographic information and bone measures and correlations between each of the baseline bone measures.

## Materials and Methods

### Study design and participants

This study presents data collected as an ancillary project to a recently completed clinical trial, the Cooperative Lifestyle Intervention Program II (CLIP II; NCT01547182). Study design and methods [19] and treatment effects on mobility and muscle strength (main outcomes) are published [20]. Briefly, the parent study included 252 older (60-79 years), overweight and obese (BMI ≥ 28 kg/m^2^) adults who had cardiovascular disease or metabolic syndrome and self-reported mobility disability, and was designed to evaluate the effects of diet-induced weight loss and exercise on mobility and lower-body strength. Clinically meaningful (i.e. ≥5% baseline weight) weight loss was achieved in all treatment groups. A subset of participants from the Clip II trial were recruited and consented to participate in the present study. Participants had a hip CT scan at baseline (n=55) and 18-month follow-up (n=34). 21 participants (38%) did not have a post-intervention CT scan (6 participants completed the intervention but did not have a post-intervention CT scan and 15 participants dropped out of the study).

### CT imaging and processing

CT scans of the hip (120 kVp, 350 mA, 500 mm field of view, 0.977 mm pixel size and 1.25 mm slice thickness acquired helically with a pitch of 1.375:1, standard reconstruction with secondary reconstruction to 0.625 mm slice thickness) covering the region from the superior acetabulum to the mid-femur were acquired using a 64-slice CT scanner (LightSpeed VCT, General Electric Medical Systems, Milwaukee, WI). Image segmentation of the femur was performed using Mimics software (Materialise, Plymouth, MI) using automated operations including thresholding and region growing techniques and manual editing. Results of the segmentation include binary images and masks of both the left and right subject femurs. The segmentation masks were converted into 3D triangulated surface models of the femur.

### Volumetric bone mineral density measurement

**Figure 1:**
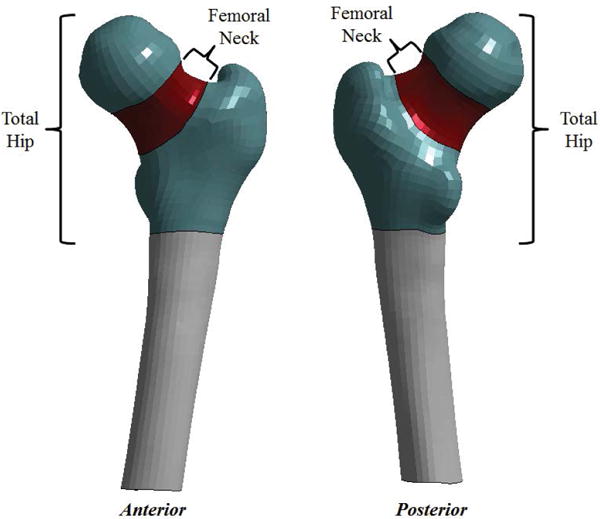
Definition of total hip and femoral neck regions for the proximal femur shown in the anterior and posterior view.

vBMD of the total hip and femoral neck were obtained using N-vivo software (Image Analysis, Columbia, KY). The software enables automated 3D segmentation of the hip that divides the proximal femur into anatomical compartments. The CT Hounsfield units (HU) are calibrated using a 4-port In Table bone mineral phantom to derive equivalent calcium hydroxyapatite density measures or vBMD in g/cm^3^. This method was applied to the total hip region of interest as well as the femoral neck region of interest to derive integral vBMD measures for both regions. A depiction of the definition of the total hip and femoral neck regions is provided in Figure 1.

Elasticity-density relationships from the literature were used to derive subject-specific material properties for the FE models for the total hip and femoral neck regions. Ash density (ρ_ash_, g/cm^3^) of the total hip and femoral neck was computed from the calibrated vBMD (ρ_CT_, g/cm^3^) and normalized to an apparent density (ρ_app_, g/cm^3^) using Equations 1 and 2 [21]. Elastic modulus (E, GPa) was calculated from the elasticity-density relationship reported by Morgan et al. which has been shown to produce accurate results in strain prediction for subject-specific FE models [17,22] using Eq. 3. The elastic modulus derived from the integral vBMD was assigned to the cortical shell for the total hip and femoral neck regions for baseline and 18-month post-intervention follow-up. The elastic modulus of the trabecular bone remained constant across all patients with the given baseline model parameters [23].

(1) $$\rho_{\mathrm{ash}}=0.877*\rho_{CT}+0.079$$

(2) $$\rho_{\mathrm{app}}=\frac{\rho_{\mathrm{ash}}}{0.6}$$

(3) $$E=6.85*\rho_{\mathrm{app}}{\;}^{1.49}$$

### Cortical thickness assessment

A cortical thickness estimation algorithm was applied to the proximal femur to derive variable cortical thickness values across the entire surface (Stradwin v5.2, Cambridge University, UK) [24,25]. Cortical thickness mapping relies on a global cortical density measurement from the CT data which is factored into a piecewise defined Heaviside step function derived from the convolution of both in-plane and out-of-plane point spread functions [26]. HU values along a profile line normal to the femoral cortical surface were fit to a mathematical model constrained by the cortical density and out-of-plane blur, to obtain approximately 14,000 cortical thickness measurements from each femur. Algorithm outputs include point clouds of the inner and outer surfaces as well as a calculated thickness. Cortical thickness of the proximal femur was collected at baseline and 18-month post-intervention follow-up.

**Figure 2:**
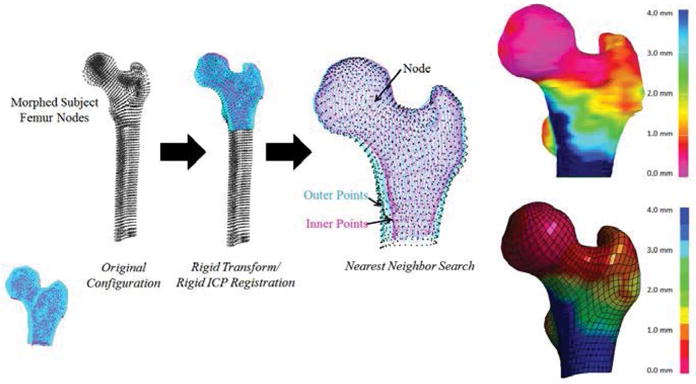
Cortical thickness mapping workflow. [Above, it shows the transformations and nearest neighbor search processes and on the right, the results of the cortical thickness mapping. The output of the cortical thickness estimation algorithm for an example subject is shown on top right with the resulting subject-specific FE model with cortical thickness mapped on the bottom right.]

To incorporate the cortical thickness maps into the FE models, a mapping approach was developed to assign each node of the cortical shell a variable cortical thickness value. Following the alignment of the cortical thickness point cloud and FE models, custom Matlab code was used to perform a nearest neighbor search for each FE node and assign the thickness value based on the output of the cortical thickness estimation algorithm. The cortical thickness mapping process and results for an example femur are shown in Figure 2.

### Finite element model development

Subject-specific FE models of the proximal femur were developed using morphing techniques to accelerate the development of the models as described by previous literature [27–30]. The morphing procedure involves the use of radial basis function interpolation using the thin-plate spline basis function and a relaxation algorithm to morph an existing FE model to a subject-specific geometry [31–33]. The thin-plate spline algorithm uses homologous landmark data in a reference and target configuration to derive an interpolation function and coefficients which can be applied to the nodal coordinates of the reference FE model as it is associated with the reference homologous landmark data. Homologous landmark data are points on analogous positions on each respective geometry that allow for a one-to-one mapping between the reference and target configurations. The proximal femur of the Global Human Body Models Consortium (GHBMC) v4.4 average male occupant model was used for the reference homologous landmark data and FE model. The GHBMC M50 FE model is representative of a 50^th^ percentile male (M50) and was based on medical images of a 26 year old individual (height, 174.9 cm; weight, 78.6 kg; and body mass index (BMI), 25.7) [34–36]. The femur model of the GHBMC M50 has been validated against post-mortem human surrogate (PMHS) data in various loading conditions including quasi-static compression in stance and fall configurations, three-point bending of the femoral shaft, and dynamic combined bending and compression loading [23]. Overall, the model showed good agreement with PMHS data and therefore was selected as the reference model for this study due to its biofidelity and robustness. The target homologous landmark data was derived from the subject-specific CT data of the proximal femur.

**Figure 3:**
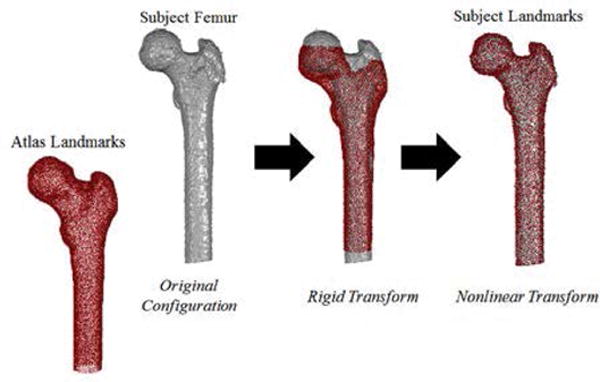
Registration of atlas landmarks (red) to the subject segmentations (grey) using rigid and nonlinear transformations for the femur to generate subject landmarks.

Homologous landmark collection of the reference and subject-specific femurs utilized image segmentation, atlas development, and image registration techniques [37,38]. An image registration algorithm was applied using rigid and nonlinear transformations to register the binary images of the atlas femur to the binary images of the segmented subject-specific femur to map the atlas landmarks of the femur to each subject-specific femur (Figure 3).

**Figure 4:**
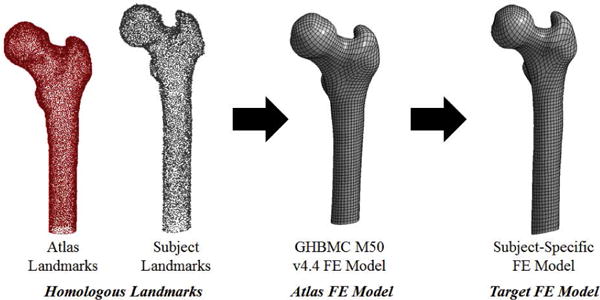
Model morphing of GHBMC M50 v4.4 femur to a subject-specific model. Homologous landmarks of the atlas (red) and subject (grey) are used to derive an interpolation function that morphs the atlas FE model to the target or subject-specific model.

The outputs of the image registration process are the homologous landmarks for the subject-specific femurs. These serve as the inputs to the model morphing procedure in addition to the atlas landmarks and the FE nodal coordinates. The thin-plate spline morphing process for the femur is depicted in Figure 4. A deviation analysis was performed to measure the point-to-surface distances to evaluate the quality and robustness of the image registration algorithm and thin-plate spline morphing algorithm (See Supplemental Appendix).

### Finite element model simulation

Bone strength was estimated through simulation of a single-limb stance and sideways fall configuration involving quasi-static compression of the femoral head and neck [13,15]. The published experimental data for both the stance and fall configuration was based on 18 human cadaveric proximal femurs (10 F+8 M) with an average age of 70.3 years old (range: 52-92 years). The atlas GHBMC M50 v4.4 femur was validated against the published experimental data for each configuration to evaluate the biofidelity (See Supplemental Appendix). To analyze baseline bone measures, subject-specific FE models were developed for 48 participants who had complete bone measure data (vBMD, cortical thickness, and bone strength) for the baseline scans. Missing baseline bone measures were due to missing bone mineral phantom ports (n=2), failure of N-vivo software to detect the hip (n=4), and a patient with a total hip replacement (n=1). To analyze the 18-month change in weight and bone measures, subject-specific FE models were developed for 25 participants who had complete bone measure data for both baseline and follow-up. For the 34 participants with both baseline and follow-up CT scan data, baseline and 18-month post-intervention bone measure data was obtained for 25 participants. Missing baseline and follow-up bone measure data was due to failure to scan the region of interest with the femoral head cutoff (n=5), missing bone mineral phantom ports (n=2), and failure of N-vivo software to detect the hip (n=2). A total of 192 simulations were performed for the developed baseline models (Table 1). The simulations included 2 loading configurations (stance and fall), 2 structures (left and right femur) and 1 model per subject (baseline) for the 48 participants. A total of 200 simulations were performed for the developed baseline and follow-up models (Table 1). The simulations included 2 loading configurations (stance and fall), 2 structures (left and right femur), and 2 models per subject (baseline and 18-month post-intervention) for the 25 participants. All simulations were performed using nonlinear implicit analysis with LS-Dyna (v6.0.0 rev 71482, LSTC, Livermore, CA).

**Figure 5:**
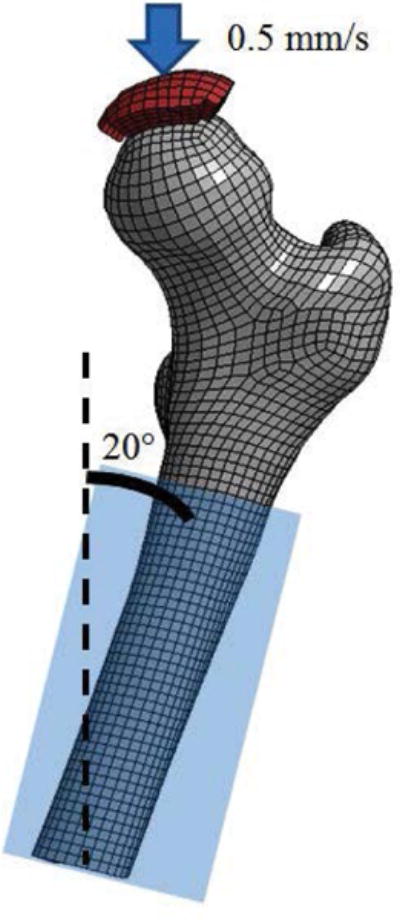
Simulation setup for the single-limb stance configuration.

**Figure 6:**
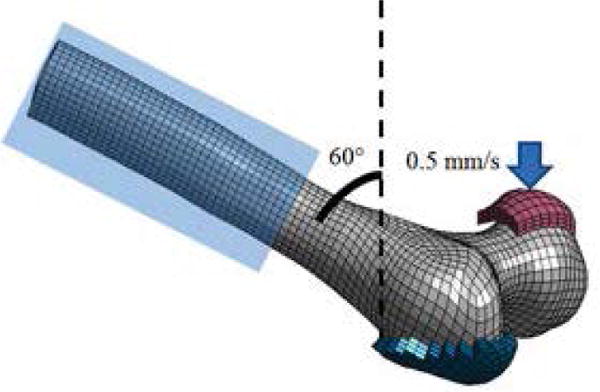
Simulation setup for the sideways fall configuration.

For the single-limb stance configuration, the femoral longitudinal axis was positioned at a 20° angle to the vertical axis in the coronal plane with the femoral shaft fully constrained (Figure 5). A 30 mm diameter molded polymethylmethacrylate (PMMA) impactor compressed the femoral head at a rate of 0.5 mm/s until fracture. For the sideways fall configuration, the femoral longitudinal axis was positioned at a 60° angle to the vertical axis and at a 70° angle to the major axis of the elliptical cross-section of the femoral neck (Figure 6). The femoral shaft was fully constrained and the greater trochanter was placed onto a molded PMMA cup as described in the experimental setup. Similar to the stance configuration, a 30 mm diameter molded PMMA impactor compressed the femoral head at a rate of 0.5 mm/s until fracture. The peak fracture force was defined as the peak force recorded between the impactor and femoral head for both configurations. The proximal femur was modeled as an isotropic elastic-plastic material with a failure strain. Bone fracture is predicted using the element deletion method that removes elements when a given strain threshold is exceeded. For the proximal femur, cortical bone fracture was modeled with a fracture threshold of 0.0088 effective plastic strain [23,39].

### Baseline covariate assessments

Participant age, gender, and ethnicity were captured via self-report at the baseline assessment visit. Additional information on prior fracture, parent fracture, smoking status, glucocorticoid use, alcohol use, and diagnosis of rheumatoid arthritis were collected via self-report and used to calculate the 10-year probability of major osteoporotic and hip fracture using the FRAX^®^ tool (https://www.shef.ac.uk/FRAX/tool.jsp). Height was assessed without shoes to the nearest 0.25 cm using a stadiometer (HealthO Meter^®^ Portrod) and body mass measured to the nearest 0.05 kg using a calibrated and certified digital scale (HealthO Meter^®^ Professional 349KLX). aBMD (g/cm^2^) was assessed using DXA (iDXA, GE Medical Systems, Madison, WI) at baseline at the posterior-anterior lumbar spine (L1-L4) and hip (femoral neck, trochanter, and intertrochanteric space) using the manufacturer’s recommendations for patient positioning and scanning. Osteoporosis and osteopenia were defined as location-specific T-scores ≤2.5 and between −2.5 and −1, respectively [40].

### Statistical analysis

Descriptive statistics were calculated overall and by gender at baseline among all participants with baseline bone measurements, and gender and ethnicity group comparisons of baseline bone measures were performed using t-tests. Associations between bone measures and continuous patient characteristics (age, BMI, and body mass) as well as correlations among bone measures were performed using both Pearson correlations and simple linear regression. Changes at 18-months used only participants with both baseline and 18-month data, and comparisons of participants with and without follow-up data were performed using t-tests for continuous variables and chi-square tests for discrete characteristics. One-sample t-tests were used to test the differences in weight and bone measure data across the 18-months. The associations between changes in bone measures were analyzed using Pearson correlations and simple linear regression. Further associations between changes in bone measures and changes in body mass were measured using partial Pearson correlations and multiple linear regression models adjusted for gender and baseline bone measure and body mass data. Due to the hypothesis-generating nature of these analyses there were no adjustments for multiple comparisons. All statistical analyses were performed using SAS v9.4 (SAS Institute, Cary, NC) and a p-value less than 0.05 was considered statistically significant.

## Results

### Baseline demographic characteristics

Fifty-five participants completed pre-intervention assessments and commenced the intervention. Baseline characteristics of the 55 participants are included in Table 2, including descriptive statistics of age, weight, BMI, ethnicity, FRAX score, and clinical categorization of bone health. Briefly, participants were 65.8 ± 4.3 years of age, with the majority being female (64%) and Non-Hispanic white (69%). Body mass was 96.1 ± 16.9 kg and BMI was 34.0 ± 3.5 kg/m^2^ with 53% of the study sample classified as Type I Obese. Based on DXA, T-scores indicated normal bone density in 36% of the participants, osteopenia in 53% of participants, and osteoporosis in 11% of participants. Based on the FRAX score, 10-year probability of major osteoporotic fracture or hip fracture was low.

### Baseline bone measure summary

Descriptive statistics of the full baseline sample (n=55) where bone measure data was available (n=48-54) are summarized in Table 3. Overall, the mean vBMD of the femoral neck was greater than the mean of the total hip. The mean cortical thickness of the total hip was greater than the mean of the femoral neck. Mean estimated strength values for both femoral stance and femoral fall were within the range of the published experimental testing for each configuration [13,15]. As a sensitivity analysis, descriptive data were stratified by gender and ethnicity (data not shown). There were no statistically significant differences in vBMD or cortical thickness measures between men or women or between participants of different ethnicities (all p>0.05); although, baseline proximal femur strength in the stance and fall configuration was significantly greater for the men in comparison to the women (p <0.001 stance; p=0.002 fall).

Correlations and parameter estimates between continuous baseline patient characteristics and baseline bone measures are listed in Table 4. Baseline proximal femur strength in the stance and fall configurations significantly decreases with age (p=0.04 stance; p=0.003 fall). For baseline BMI, a significant positive correlation was found with baseline cortical thickness for the total hip (r=0.40, p=0.05) and femoral neck (r=0.43, p=0.03). For baseline body mass, a significant positive correlation was found with baseline femoral stance strength (r=0.63, p<0.001) and baseline femoral fall strength (r=0.49, p=0.01).

Correlations and parameter estimates between each of the baseline bone measures are listed in Table 5. Baseline vBMD of the total hip was positively correlated with baseline cortical thickness of the total hip (r=0.57, p=0.003). Baseline vBMD of the femoral neck was positively correlated with baseline cortical thickness of the femoral neck (r=0.68, p<0.001). A negative correlation between baseline vBMD and femoral strength was noted. Significant correlations between baseline vBMD of the total hip and femoral neck with femoral stance and fall strength are likely confounded by other baseline variables including body mass and gender. Baseline femoral stance strength was positively correlated with baseline femoral fall strength (r=0.85, p<0.001).

### 18-month change in weight and bone measure summary

Descriptive statistics of the 25 participants (65.6 ± 4.1 average age, 76% female, 20% African American, BMI 29.7 ± 4.0 kg/m2) with baseline and 18-month post-intervention body mass and bone measures are summarized in Table 6. No significant differences in age, gender, ethnicity, or BMI were observed between the full baseline sample (n=55) and those with follow-up data (n=25; all p>0.05). Overall loss of body mass (−8.74 ± 6.64 kg) was statistically significant from baseline to 18-month post-intervention (p<0.01). Statistically significant decreases in vBMD and cortical thickness of the total hip and femoral neck as well as estimated strength for both the stance and fall configurations were observed (all p<0.01). Specifically, vBMD of the total hip and femoral neck decreased by 8.0% and 3.9%, respectively. Cortical thickness of the total hip and femoral neck decreased by 2.1% and 1.4%, respectively. Estimated strength for the stance and fall configuration decreased by 2.5% and 2.1%, respectively.

Correlations and parameter estimates between the changes of each of the bone measures are listed in Table 7. Change in vBMD of the femoral neck was positively correlated with the change in femoral fall strength (r=0.45, p=0.02). Correlations and parameter estimates between change in body mass and change in bone measures are presented in Table 8. Unadjusted correlations ranged from −0.29 to 0.56 and remained largely unchanged after adjustment for baseline bone measures, baseline body mass, and gender (r= −0.42 to 0.57). Adjusted analyses show a significant correlation between change in body mass and change in cortical thickness (total hip, r=0.56, p=0.007; femoral neck, r=0.57, p=0.006). For every 1 kilogram of body mass lost, cortical thickness in the total hip and femoral neck decreased by 0.003 mm and 0.004 mm, respectively. No significant correlations were present for the vBMD or strength data.

## Discussion

In this study, we developed subject-specific FE models of the proximal femur of overweight and obese, older adults undergoing intentional weight loss using methods which incorporate subject-specific geometry, vBMD-derived material properties, and variable cortical thickness to model both cortical and trabecular bone. Previous studies of CT-based FE models of the proximal femur have implemented voxel-based meshing techniques which produce irregular boundaries, lack the distinction between cortical and trabecular bone, and result in inaccuracies in predicting failure location [11,13–16]. While these methods are fully automatic, voxel-based FE models of the proximal femur have been shown to produce inaccuracies especially for the predicted stresses and strains on the bone surface [41,42]. The model morphing technique used in this study takes advantage of using a validated hexahedral meshed proximal femur atlas FE model to derive the subject-specific FE models. These techniques presented allow for characterization of geometry, cortical thickness, and material properties which can ultimately provide FE models that better predict bone strength and fracture risk.

Novel data generated here confirm and extend prior literature suggesting weight loss in older adults is associated with reduced total hip and femoral neck BMD and cortical thickness. Analysis of the 18-month change in bone measure data revealed statistically significant decreases from baseline to 18-months post weight loss intervention in vBMD (total hip: 8.0%; femoral neck: 3.9%), cortical thickness (total hip: 2.1%; femoral neck: 1.4%), and estimated strength (stance: 2.5%; fall: 2.1%). Emerging data implicate weight and weight change as well as BMD in fracture risk prediction. Previous studies have found significant decreases in total hip aBMD associated with diet-induced weight loss which can increase fracture risk [43]. In this meta-analysis, 0.010 to 0.015 g/cm^2^ decreases in aBMD associated with diet-induced weight loss were similar to the average yearly aBMD loss for elderly women [1,43,44]. With each standard deviation decrease in femoral neck aBMD of 0.12 g/cm^2^, risk of hip fracture is approximately increased by 3.5 fold [45]. In our study, participants lost an average of 0.012 g/cm^3^ in the femoral neck integral vBMD. For a standard deviation decrease in estimated femoral neck vBMD of 0.05 g/cm^3^, risk of hip fracture increased by 3.3 fold [46]. Therefore, for the population presented in this study, the decrease in femoral neck vBMD would result in an approximately 0.8-fold change in fracture risk across the 18-month intervention. In analyses adjusted for baseline bone measures, baseline body mass, and gender, significant correlations were found between weight change and change in total hip and femoral neck cortical thickness. In order to identify optimal weight loss therapies that minimize bone loss with weight loss, it is important to measure the change in bone density and quality including cortical thickness and vBMD as described in this study to further understand the effects of weight loss on bone health in older adults.

Analysis of baseline bone measures revealed for baseline BMI, a significant positive correlation with cortical thickness and negative correlation with baseline femoral stance and fall strength. These results suggest that while baseline cortical thickness is thicker with higher BMIs, the baseline femoral strength is weaker which indicates bone strength is not explained only by the thickness. Bone strength is a function of geometry, cortical thickness, and material properties. Therefore, thicker bones in an older, obese adult population may not necessarily result in stronger bones due to lower vBMD values. In addition, a significant positive correlation was found between baseline body mass and estimated strength indicating that greater body mass is associated with increased estimated strength values due in part to the increased mechanical loads on the hip.

No statistically significant correlations were present for change in body mass and change in vBMD or bone strength. One potential explanation is that bone strength factors in the redistribution of cortical thickness across the proximal femur which has been shown to be localized following exercise-induced changes where mechanical demands are the greatest [47]. In addition, with weight loss there are reductions in mechanical loads which could induce changes in the distribution of the cortical thickness which can ultimately affect the estimated bone strength. As a note, the sample of 25 participants with baseline and 18-month post-intervention data represents a pilot data set and additional data would be necessary to draw further conclusions on change in body mass and bone strength. Bone strength is a function of geometry, cortical thickness, and vBMD. The relative contribution and change of each of the components (geometry, cortical thickness, and vBMD) with change in body mass could vary by participant. We hypothesize, with more data, that there would be significant changes in vBMD and bone strength with weight loss.

Previous studies of FE estimated proximal femur strength have shown reduced strength is associated with incidents of hip fracture with men and women having 20-31% lower strength in the stance configuration and 29-38% lower strength in the fall configuration, when comparing subjects with fracture with age and sex-matched controls [48]. Controlling for aBMD, the association between hip fracture risk and estimated FE strength remained, suggesting that estimated FE strength accounts for characteristics of the proximal femur that cannot be captured by BMD alone. In the study presented, the significant reductions in estimated strength from baseline to 18-month post-intervention following weight loss in both the stance and fall configuration may increase hip fracture risk. In addition, reductions in cortical thickness and vBMD are also associated with increases in hip fracture risk [8,12]. Future studies can develop logistic regression models to incorporate the effects of bone strength, cortical thickness, and vBMD to determine the probability of hip fracture for a given age and gender. Hip fracture risk is also dependent on the risk of falling; integration of FE estimated strength with assessment of fall risk could improve the accuracy of fracture risk prediction.

There are a number of limitations associated with this study. In terms of model development, one limitation is the use of homogenous material properties within each of the total hip and neck regions. Studies have shown more accurate results for inhomogeneous material properties on an element-by-element basis so there is potential to improve the models developed in this study by deriving a vBMD measure for each element. In addition, the use of integral vBMD to assign cortical bone material properties, does underestimate the elastic modulus. The elastic modulus values assigned to the cortical bone for the neck ranged from ~2.2-4.5 GPa and for the total hip ranged from ~2.4-3.8 GPa. The baseline GHBMC femur assigns values of 2 GPa and 6 GPa to the head and neck cortical regions, respectively [23]. These values were determined based on the literature which found that the femoral head subchondral bone in the weight-bearing region is approximately 1.5 GPa [49] and that the cortical shell in the femoral neck region is 24% less than the shaft region (shaft region range: 6-21 GPa) [39,50,51]. The values assigned to the models were in the range of literature and the strength estimates fell within the experimental range, so the use was deemed suitable at this time. Future work includes techniques to obtain cortical and trabecular vBMD measures [52].

A limitation is also present for the collection of cortical thickness data using the cortical thickness estimation algorithm. The algorithm was validated within 0.3 mm at a resolution of 0.589 mm/pixel. The resolution of the scans presented in this study was 0.977 mm/pixel. In the literature, the cortical thickness algorithm used in this study has been applied to a case-cohort of older men with a resolution of 0.94 mm/pixel [53–55]. While the accuracy of the method is not quantified for these lower scan resolutions, future work could further validate the thickness limit. This work is beyond the scope of the current study.

There also exist some limitations associated with the validation of the atlas FE model. The model response was compared to peak fracture force data reported in the literature. Future work includes further validation using force-time or force-displacement data when the experimental data becomes available. In order to improve the validation of the atlas model and fully validate the subject-specific models, the techniques developed in this study must be validated against controlled cadaveric experiments to establish a prediction accuracy of the modelling technique. The focus of this study was on the change in bone parameters including estimated strength using the developed FE models. Therefore, while the subject-specific FE strength data falls within range of the published experimental data, the focus should not be on the exact value predicted but rather the change in bone strength in order to estimate effects of weight loss. Another factor related to the FE models includes the fracture criteria. While strain-based failure criteria and element deletion are generally accepted to model fracture, more accurate techniques should be investigated to better model fracture initiation and propagation. The simulation configurations for the stance and fall also neglect soft tissue effects. Studies have shown the protective effect of obesity and excess adipose tissue in reducing forces transferred to the femur [3]. The focus of this study was to study the effects of weight loss on bone measures. Future work could improve simulation configurations to include surrounding soft tissue to estimate bone strength.

In conclusion, the subject-specific FE models of the proximal femur of overweight and obese, older adults developed in this study were employed to improve the understanding of intentional weight loss on bone measures. The methods presented in this study offer the ability to accurately account for variations in geometry, cortical thickness, and material properties which can result in more reliable FE models to predict bone strength. The techniques used in this study can be applied to a larger sample population to improve the understanding of the complex relationship between morphologic, compositional, and material changes of older adults who are overweight or obese undergoing intentional weight loss. These tools, in addition to aBMD measures, can be used further understand the effects of intentional weight loss on bone health and aid clinicians in designing optimal weight loss strategies for older persons.

## Supplementary Material

Supplemental Appendix

## Figures and Tables

**Table 1:** Simulation test matrix for baseline (n=48) and baseline and follow-up (n=25) models.

Loading Configuration	#of Structures	Model	N Patients	N Simulations
**Stance**	2	Baseline (1)	48	96
		Baseline + Follow-up (2)	25	100
**Fall**		Baseline (1)	48	96
		Baseline + Follow-up (2)	25	100
**Total**				392

**Table 2:** Mean (SD) baseline characteristics of study population.

Variable	Men (n=20)	Women (n=35)	Overall (n=55)
Age (years)	65.6 ± 4.4	66.0 ± 4.3	65.8 ± 4.3
Body mass (kg)	109.2 ± 16.8	88.6 ± 11.6	96.1 ± 16.9
BMI (kg/m^2^)	34.3 ± 3.8	33.8 ± 3.5	34.0 ± 3.5
African American, n (%)	5 (25)	11(31)	16 (29)
**FRAX 10-year probability (%)**			
Major Fracture	3.9 ± 2.9	4.2 ± 2.2	4.1 ± 2.4
Hip Fracture	0.05 ± 0.09	0.04 ± 0.08	0.04 ± 0.08
**Clinical Categorization, n (%)**			
Normal	6 (30)	14 (40)	20 (36)
Osteopenia	10 (50)	19 (54)	29 (53)
Osteoporosis	4 (20)	2 (6)	6 (11)

**Table 3:** Baseline bone measure descriptive data.

	Baseline	
	N	Mean ± SD
**vBMD (g/cm^3^)**		
Total Hip	48	0.301 ± 0.036
Femoral Neck	48	0.313 ± 0.043
**Cortical Thickness (mm)**		
Total Hip	54	2.030 ± 0.238
Femoral Neck	54	1.871 ± 0.242
**Estimated Strength (kN)**		
Femoral Stance	48	6.18 ± 1.00
Femoral Fall	48	2.07 ± 0.39

**Table 4:** Correlations and parameter estimates between baseline bone measures and baseline patient characteristics.

Bone Measure	Age (years)		BMI (kg/m^2^)		Body Mass (kg)	
	r	β(SE)	r	β (SE)	r	β (SE)
**vBMD (g/cm^3^)**						
Total Hip	0.51*	0.004 (0.001)	0.29	0.002 (0.002)	−0.18	0.000 (0.001)
Femoral Neck	0.47*	0.005 (0.002)	0.23	0.002 (0.002)	−0.22	−0.001 (0.001)
**Cortical Thickness (mm)**						
Total Hip	0.26	0.014 (0.011)	0.40*	0.024 (0.011)	0.31	0.006 (0.004)
Femoral Neck	0.27	0.013 (0.010)	0.43*	0.022 (0.010)	0.23	0.004 (0.004)
**Estimated Strength (kN)**						
Femoral Stance	−0.41*	−0.101 (0.046)	−0.27	−0.068 (0.051)	0.63*	0.056 (0.014)
Femoral Fall	−0.57*	−0.046 (0.014)	−0.33	−0.028 (0.017)	0.49*	0.014 (0.005)

* *p*-value<0.05.

**Table 5:** Correlations and parameter estimates between baseline bone measures.

Region	Bone Measure Correlations	r	β (SE)
**Total Hip**	vBMD (g/cm^3^) & Cortical Thickness (mm)	0.57*	4.278 (1.296)
	vBMD (g/cm^3^) & Femoral Stance Strength (kN)	−0.37	−12.151 (6.368)
	vBMD (g/cm^3^) & Femoral Fall Strength (kN)	−0.47*	−5.092 (2.012)
	Cortical Thickness (mm) & Femoral Stance Strength (kN)	−0.02	−0.094 (0.908)
	Cortical Thickness (mm) & Femoral Fall Strength (kN)	−0.17	−0.251 (0.297)
**Femoral Neck**	vBMD (g/cm^3^) & Cortical Thickness (mm)	0.68*	3.240 (0.734)
	vBMD (g/cm^3^) & Femoral Stance Strength (kN)	−0.47*	−11.208 (4.403)
	vBMD (g/cm^3^) & Femoral Fall Strength (kN)	−0.58*	−4.631 (1.344)
	Cortical Thickness (mm) & Femoral Stance Strength (kN)	−0.15	−0.734 (1.031)
	Cortical Thickness (mm) & Femoral Fall Strength (kN)	−0.37	−0.621 (0.321)
	Femoral Stance Strength (kN) & Femoral Fall Strength (kN)	0.85*	0.281 (0.037)

* *p*-value<0.05.

**Table 6:** Baseline and 18-month post-intervention body mass and bone measure descriptive data (n=25).

	Baseline Mean ± SD	18-month Mean ± SD	Change (95% CI)	*p*-value
**Body Mass (kg)**	89.14 ± 11.40	80.40 ± 11.33	−8.74 (−11.84, −5.99)	<0.0001*
**vBMD (g/cm^3^)**				
Total Hip	0.299 ± 0.031	0.275 ± 0.033	−0.024 (−0.029, −0.019)	<0.0001*
Femoral Neck	0.308 ± 0.042	0.296 ± 0.047	−0.012 (−0.018, −0.006)	0.0002*
**Cortical Thickness (mm)**				
Total Hip	2.064 ± 0.232	2.020 ± 0.233	−0.044 (−0.057, −0.030)	<0.0001*
Femoral Neck	1.878 ± 0.202	1.852 ± 0.198	−0.026 (−0.042, −0.011)	0.002*
**Estimated Strength (kN)**				
Femoral Stance	6.11 ± 1.01	5.96 ± 1.00	−0.15 (−0.20, −0.10)	<0.0001*
Femoral Fall	2.04 ± 0.34	2.00 ± 0.33	−0.04 (−0.07, −0.02)	0.002*

*p*-values indicate if change from baseline to 18-months is significant

* *p*-value<0.05

**Table 7:** Correlations and parameter estimates between changes in bone measure.

Region	Bone Measure Correlations	r	β (SE)
**Total Hip**	Δ vBMD (g/cm^3^) & Δ Cortical Thickness (mm)	0.15	0.385 (0.527)
	Δ vBMD (g/cm^3^) & Δ Femoral Stance Strength (kN)	−0.25	−2.391 (1.920)
	Δ vBMD (g/cm^3^) & Δ Femoral Fall Strength (kN)	−0.16	−0.796 (1.052)
	Δ Cortical Thickness (mm) & Δ Femoral Stance Strength (kN)	0.12	0.434 (0.770)
	Δ Cortical Thickness (mm) & Δ Femoral Fall Strength (kN)	0.00	−0.002 (0.416)
**Femoral Neck**	Δ vBMD (g/cm^3^) & Δ Cortical Thickness (mm)	0.23	0.653 (0.568)
	Δ vBMD (g/cm^3^) & Δ Femoral Stance Strength (kN)	0.29	2.556 (1.739)
	Δ vBMD (g/cm^3^) & Δ Femoral Fall Strength (kN)	0.45*	2.122 (0.870)
	Δ Cortical Thickness (mm) & Δ Femoral Stance Strength (kN)	0.18	0.550 (0.639)
	Δ Cortical Thickness (mm) & Δ Femoral Fall Strength (kN)	0.09	0.143 (0.347)
	Δ Femoral Stance Strength (kN) & Δ Femoral Fall Strength (kN)	0.25	0.133 (0.108)

* *p*-value<0.05.

**Table 8:** Correlations and parameter estimates between change in body mass (kg) and change in bone measures, unadjusted and adjusted for baseline bone measures, body mass and gender.

	Δ Body Mass (kg)			
	Unadjusted		Adjusted	
**Δ Bone Measure**	r	β (SE)	r	β (SE)
**Δ vBMD (g/cm^3^)**				
Total Hip	0.2	0.000 (0.000)	0.23	0.000 (0.000)
Femoral Neck	−0.04	0.000 (0.000)	−0.1	0.000 (0.000)
**Δ Cortical Thickness (mm)**				
Total Hip	0.56*	0.003 (0.001)	0.56*	0.003 (0.001)
Femoral Neck	0.43*	0.003 (0.001)	0.57*	0.004 (0.001)
**Δ Strength (kN)**				
Femoral Stance	0.06	0.001 (0.004)	0.01	0.000 (0.005)
Femoral Fall	−0.29	−0.003 (0.002)	−0.42	−0.004 (0.002)

## References

[1] Ensrud KE, Ewing SK, Stone KL, Cauley JA (2003). Intentional and unintentional weight loss increase bone loss and hip fracture risk in older women. J Am Geriatr Soc.

[2] Ensrud KE, Fullman RL, Barrett-Connor E, Cauley JA, Stefanick ML (2005). Voluntary weight reduction in older men increases hip bone loss: the osteoporotic fractures in men study. J Clin Endocrinol Metab.

[3] Shapses SA, Sukumar D (2012). Bone metabolism in obesity and weight loss. Annu Rev Nutr.

[4] Nguyen ND, Eisman JA, Center JR, Nguyen TV (2007). Risk factors for fracture in nonosteoporotic men and women. J Clin Endocrinol Metab.

[5] Tothill P (2005). Dual-energy x-ray absorptiometry measurements of total-body bone mineral during weight change. J Clin Densitom.

[6] Hangartner TN, Johnston CC (1990). Influence of fat on bone measurements with dual-energy absorptiometry. Bone Miner.

[7] Poole KE, Treece GM, Mayhew PM, Vaculík J, Dungl P (2012). Cortical thickness mapping to identify focal osteoporosis in patients with hip fracture. PLoS One.

[8] Johannesdottir F, Poole KE, Reeve J, Siggeirsdottir K, Aspelund T (2011). Distribution of cortical bone in the femoral neck and hip fracture: a prospective case-control analysis of 143 incident hip fractures; the AGES-REYKJAVIK Study. Bone.

[9] Link TM (2012). Osteoporosis imaging: state of the art and advanced imaging. Radiology.

[10] Engelke K, Lang T, Khosla S, Qin L, Zysset P (2015). Clinical use of quantitative computed tomography (QCT) of the hip in the management of osteoporosis in adults: the 2015 ISCD official positions—part I. J Clin Densitom.

[11] Cody DD, Gross GJ, Hou FJ, Spencer HJ, Goldstein SA (1999). Femoral strength is better predicted by finite element models than QCT and DXA. J Biomech.

[12] Lang T, Keyak JH, Heitz MW, Augat P, Lu Y (1997). Volumetric quantitative computed tomography of the proximal femur: precision and relation to bone strength. Bone.

[13] Keyak JH, Rossi SA, Jones KA, Skinner HB (1998). Prediction of femoral fracture load using automated finite element modeling. J Biomech.

[14] Keyak J (2001). Improved prediction of proximal femoral fracture load using nonlinear finite element models. Med Eng Phys.

[15] Keyak JH, Rossi SA, Jones KA, Les CM, Skinner HB (2001). Prediction of fracture location in the proximal femur using finite element models. Med Eng Phys.

[16] Keyak J, Sigurdsson S, Karlsdottir GS, Oskarsdottir D, Sigmarsdottir A (2013). Effect of finite element model loading condition on fracture risk assessment in men and women: the AGES-Reykjavik study. Bone.

[17] Schileo E, Taddei F, Malandrino A, Cristofolini L, Viceconti M (2007). Subject-specific finite element models can accurately predict strain levels in long bones. J Biomech.

[18] Schileo E, Taddei F, Cristofolini L, Viceconti M (2008). Subject-specific finite element models implementing a maximum principal strain criterion are able to estimate failure risk and fracture location on human femurs tested in vitro. J Biomech.

[19] Marsh AP, Janssen JA, Ambrosius WT, Burdette JH, Gaukstern JE (2013). The Cooperative Lifestyle Intervention Program-II (CLIP-II): design and methods. Contemp Clin Trials.

[20] Rejeski WJ, Ambrosius WT, Burdette JH, Walkup MP, Marsh AP (2017). Community weight loss to combat obesity and disability in at-risk older adults. J Gerontol A Biol Sci Med Sci.

[21] Schileo E, Dall’ara E, Taddei F, Malandrino A, Schotkamp T (2008). An accurate estimation of bone density improves the accuracy of subject-specific finite element models. J Biomech.

[22] Morgan EF, Bayraktar HH, Keaveny TM (2003). Trabecular bone modulus– density relationships depend on anatomic site. J Biomech.

[23] Untaroiu CD, Yue N, Shin J (2013). A finite element model of the lower limb for simulating automotive impacts. Ann Biomed Eng.

[24] Treece GM, Gee AH, Mayhew PM, Poole KES (2010). High resolution cortical bone thickness measurement from clinical CT data. Med Image Anal.

[25] Treece GM, Poole KM, Gee AH (2012). Imaging the femoral cortex: thickness, density and mass from clinical CT. Med Image Anal.

[26] Treece GM, Gee AH (2015). Independent measurement of femoral cortical thickness and cortical bone density using clinical CT. Med Image Anal.

[27] Vavalle NA, Schoell SL, Weaver AA, Stitzel JD, Gayzik FS (2014). Application of radial basis function methods in the development of a 95^th^ percentile male seated fea model. Stapp Car Crash J.

[28] Schoell SL, Weaver AA, Vavalle NA, Stitzel JD (2015). Age and sex-specific thorax finite element model development and simulation. Traffic Inj Prev.

[29] Klein KF, Hu J, Reed MP, Hoff CN, Rupp JD (2015). Development and validation of statistical models of femur geometry for use with parametric finite element models. Ann Biomed Eng.

[30] Schoell SL, Weaver AA, Urban JE, Jones DA, Stitzel JD (2015). Development and validation of an older occupant finite element model of a mid-sized male for investigation of age-related injury risk. Stapp Car Crash J.

[31] Bookstein FL (1997). Morphometric tools for landmark data: geometry and biology.

[32] Donato G, Belongie S (2002). Approximate thin plate spline mappings in Proc.

[33] Stayton CT (2009). Application of thin-plate spline transformations to finite element models, or, how to turn a bog turtle into a spotted turtle to analyze both. Evolution.

[34] Gayzik F, Moreno DP, Geer CP, Wuertzer SD, Martin RS (2011). Development of a full body CAD dataset for computational modeling: a multi-modality approach. Ann Biomed Eng.

[35] Vavalle NA, Moreno DP, Rhyne AC, Stitzel JD, Gayzik FS (2013). Lateral impact validation of a geometrically accurate full body finite element model for blunt injury prediction. Ann Biomed Eng.

[36] Vavalle NA, Davis ML, Stitzel JD, Gayzik FS (2015). Quantitative validation of a human body finite element model using rigid body impacts. Ann Biomed Eng.

[37] Weaver AA, Nguyen CM, Schoell SL, Maldjian JA, Stitzel JD (2015). Image segmentation and registration algorithm to collect thoracic skeleton semilandmarks for characterization of age and sex-based thoracic morphology variation. Comput Biol Med.

[38] Weaver AA, Schoell SL, Stitzel JD (2014). Morphometric analysis of variation in the ribs with age and sex. J Anat.

[39] Untaroiu C, Darvish K, Crandall J, Deng B, Wang JT (2005). A finite element model of the lower limb for simulating pedestrian impacts. Stapp Car Crash J.

[40] World Health Organization (1994). Assessment of fracture risk and its application to screening for postmenopausal osteoporosis: report of a WHO study group.

[41] Viceconti M, Bellingeri L, Cristofolini L, Toni A (1998). A comparative study on different methods of automatic mesh generation of human femurs. Med Eng Phys.

[42] Taddei F, Cristofolini L, Martelli S, Gill HS, Viceconti M (2006). Subject-specific finite element models of long bones: an in vitro evaluation of the overall accuracy. J Biomech.

[43] Zibellini J, Seimon RV, Lee CM, Gibson AA, Hsu MS (2015). Does diet-induced weight loss lead to bone loss in overweight or obese adults? A systematic review and meta-analysis of clinical trials. J Bone Miner Res.

[44] Nguyen T, Sambrook P, Eisman J (1998). Bone loss, physical activity, and weight change in elderly women: the Dubbo Osteoporosis Epidemiology Study. J Bone Miner Res.

[45] Nguyen ND, Pongchaiyakul C, Center JR, Eisman JA, Nguyen TV (2005). Identification of high-risk individuals for hip fracture: a 14-year prospective study. J Bone Miner Res.

[46] Center JR, Nguyen TV, Pocock NA, Eisman JA (2004). Volumetric bone density at the femoral neck as a common measure of hip fracture risk for men and women. J Clin Endocrinol Metab.

[47] Allison SJ, Poole KE, Treece GM, Gee AH, Tonkin C (2015). The influence of high-impact exercise on cortical and trabecular bone mineral content and 3D distribution across the proximal femur in older men: A randomized controlled unilateral intervention. J Bone Miner Res.

[48] Keyak J, Sigurdsson S, Karlsdottir G, Oskarsdottir D, Sigmarsdottir A (2011). Male–female differences in the association between incident hip fracture and proximal femoral strength: a finite element analysis study. Bone.

[49] Brown TD, Vrahas MS (1984). The apparent elastic modulus of the juxtaricular subchondral bone of the femoral head. J Orthop Res.

[50] Lotz JC, Gerhart TN, Hayes WC (1991). Mechanical properties of metaphyseal bone in the proximal femur. J Biomech.

[51] Keller T, Mao Z, Spengler D (1990). Young’s modulus, bending strength, and tissue physical properties of human compact bone. J Orthop Res.

[52] Lang T, LeBlanc A, Evans H, Lu Y, Genant H (2004). Cortical and trabecular bone mineral loss from the spine and hip in long-duration spaceflight. J Bone Miner Res.

[53] Yang L, Burton AC, Bradburn M, Neilson CM, Orwoll ES (2012). Distribution of bone density in the proximal femur and its association with hip fracture risk in older men: the osteoporotic fractures in men (MrOS) study. J Bone Miner Res.

[54] Marshall LM, Lang TF, Lambert LC, Zmuda JM, Ensrud KE (2006). Dimensions and volumetric BMD of the proximal femur and their relation to age among older US men. J Bone Miner Res.

[55] Treece GM, Gee AH, Tonkin C, Ewing SK, Cawthon PM (2015). Predicting hip fracture type with cortical bone mapping (CBM) in the osteoporotic fractures in men (MrOS) study. J Bone Miner Res.

