# Supplementary material for: Development of Subject-Specific Proximal Femur Finite Element Models Of Older Adults with Obesity to Evaluate the Effects of Weight Loss on Bone Strength
Source: J Osteoporos Phys Act. Author manuscript; Available in PMC 2018 Apr 20. (PMC5909834; doi:10.4172/2329-9509.1000213)
Supplement: Supplemental Appendix [file NIHMS956208-supplement-Supplemental_Appendix.docx]

**APPENDIX: SUPPLEMENTAL MATERIAL**

*Deviation Analysis*

To evaluate the quality and robustness of the image registration algorithm and thin-plate spline morphing algorithm, a deviation analysis was completed to quantify the point-to-surface distances between the 3D triangulated surface models of the subject femur and either the subject-specific homologous landmarks or the morphed FE nodal coordinates, respectively. Deviation analysis was conducted using Geomagic Control (version 2015, 3D Systems, Inc., Rock Hill, SC) which computes the signed distance and absolute distance on a point-by-point basis. For both the image registration algorithm and thin-plate spline morphing algorithm, ideally the subject-specific homologous landmarks or morphed FE nodal coordinates would lie directly on the subject’s 3D triangulated surface models. The analysis focused on the percentage of deviations exceeding 1.96 mm as deviations within this range are to be expected following image registration given the scan resolution. The median in-plane scan resolution of 0.98 mm was doubled to attain the 1.96 mm deviation threshold.

For the image registration, the deviations between the subject-specific homologous landmarks and the 3D triangulated surface models of the subject femur exceeding 1.96 mm on average was 0.40%. For 95% of the femurs, less than 0.13% exhibited absolute deviations greater than 1.96 mm. These results suggest that the subject-specific homologous landmarks were successfully collected from the femurs since only a small proportion of the absolute deviations exceeded 1.96 mm. For the morphing algorithm, the deviations between the morphed FE nodal coordinates and the 3D triangulated surface models of the subject exceeding 1.96 mm on average was 0.58%. For 95% of the femurs, less than 0.19% had absolute deviations greater than 1.96 mm. Similar to the deviation analysis results for the image registration, the small proportion of deviations exceeding 1.96 mm indicates that the morphing algorithm was successful in accurately generating FE models of the subjects.

*Finite Element Model Validation*

Prior to the simulations of the developed subject-specific FE models, the atlas GHBMC M50 v4.4 femur was validated against the published experimental data for each configuration to evaluate the biofidelity. Since there was no force-time or force-displacement curves in the published experimental paper, the peak fracture force was used for comparison. The peak fracture force predicted in the FE simulations using the atlas GHBMC M50 v4.4 femur for the stance and fall configuration was 8.02 kN and 2.51 kN, respectively. The predicted FE peak fracture forces for the stance and fall were within the peak fracture forces reported in the published experimental testing of 8.44 ± 3.04 kN (range: 3.12 – 15.04 kN) and 2.38 ± 1.31 kN (range: 0.58 – 4.63 kN), respectively [1]. Therefore, these results indicate that the atlas proximal femur FE model can accurately predict bone strength in both loading configurations.

1. Keyak, J.H., et al., *Prediction of femoral fracture load using automated finite element modeling.* J Biomech, 1998. **31**(2): p. 125-133.
